# Colorectal emergencies associated with penetrating or retained foreign bodies

**DOI:** 10.1186/1749-7922-8-25

**Published:** 2013-07-13

**Authors:** Selim Yigit Yildiz, Murat Kendirci, Serkan Akbulut, Ali Ciftci, Hamdi Taner Turgut, Suleyman Hengirmen

**Affiliations:** 1 Department of General Surgery, Kocaeli Derince Training and Research Hospital, Kocaelı, Izmit, Turkey; 2 General Surgery, Edirne State Hospital, Edirne, Turkey; 3 Department of Surgical Oncology, Ankara University Medical School, Ankara, Turkey; 4 Department of General Surgery, Ankara Numune Training and Research Hospital, Ankara, Turkey

**Keywords:** Rectum, Foreign bodies, Erotica, Wounds and injuries, Operating rooms

## Abstract

**Background:**

Foreign bodies in rectum and colon is an uncommon problem in surgical practice. Anal eroticism leads amongst etiologic factors. In some patients accidents or forceful application of foreign bodies are causative factors. This study was designed to describe our experience in diagnosis and treatment of this exciting clinical problem.

**Methods:**

Data were collected prospectively from 1998 to 2013 in 30 patients. Patient demographics, diagnostic findings, location, type, extraction method, and postextraction period were reviewed.

**Results:**

All the 30 patients were their first admission in emergency service of a hospital. On admission high alcohol intake was determined in 15(50%) patients. All the patients were hospitalized. Most of the rectal foreign bodies (23 of 25) was located distal 2/3 of the rectum. Colorectal perforation was diagnosed in 5 patients who had not any retained foreign body. Under adequate anesthesia transanal extraction was implemented in 23 (92%) patients in the operating room. In the patients with proximal located rectal foreign bodies (2/25), grade III and IV rectal injury or colonic perforation (7/30) laparotomy was carried out.

**Conclusion:**

A careful physical and rectal examination is essential for correct diagnosis and localization of retained foreign bodies. Forceful and repeated efforts without sphincter relaxation is gives rise to proximal migration of objects and unwanted complications such as rectal perforation. The operating room provides adequate anaesthesia for muscle relaxation and technical advantages in transanal extraction of rectal foreign bodies. Therefore, nonoperative success rate improves. If the objects are large and proximally migrated and if the patients suffer from peritonitis due to rectal or colon perforation or pelvic sepsis, laparatomy is performed witout much delay.

## Background

The insertion of foreign bodies (FB) into the anus is an uncommon clinical problem. Most patients are present to emergency rooms when their own efforts to remove the retained object have failed [1]. The most common etiology is anal eroticism, followed by blunt or penetrating trauma by an accident or forceful action [2,3]. Because of the late admission of the patient to the hospital the management can be difficult and may be associated with the complications. This clinical review reports our experience to this rare situation and associated complications.

## Materials and methods

Clinical datas of the emergency department of Ankara Numune and Kocaeli Derince Training and Research Hospital between November 1998 and April 2013 was reviewed prospectively. Separate files was constituted for every patient on admission. Patient demographics, findings of physical examinations and the results of diagnostic and therapothic interventions were recorded. The cases of anally introduced foreign bodies and patients with a history of colorectal foreign bodies and serious symptoms were free have been included in this review. Patients with orally ingested foreign materials have been excluded. A total of 30 patients who were diagnosed with retained colorectal foreign bodies and cases with complication of forcefull access via anus.

After their medical history were taken, all the patients were evaluated in the emergency room with help of physical and rectal examination by surgeons. Abdominal and chest x-rays of each patient were taken for the localization of foreign body and to rule out pneumoperitomeum inthe case of rectal or colonic perforation. Computed tomography was performed in case of perforation and proximally located foreign bodies. Endoscopic asssesment was not carried out in the emergency room. After full evaluation, all the patients were hospitalized. Extraction of the foreign bodies were performed in the operating room. Transanal route was the first choice for extraction of rectal FB. Anaesthesia was implemented according to the need of sphincter relaxation, choice of various instruments, and laparatomy.

After the extraction procedure, rectosigmoidoscopy was performed routinely. In the patients with large and angular foreign bodies, extraction procedure which had a long duration and difficulties were controlled more carefully after extraction procedure. When traumatic rectal injuries were determined, Rectal Organ Injury Scale (ROIS) was used to classify. Rectal lesions were classified as Grade I(simple contusion) to Grade V(devascularization of rectal segment). This grade system was used to define the lesions only. Objects that can not be removed transanal route and patients with severe colorectal injuries or perforation laparotomy was carried out.

## Results

**Figure 1:**
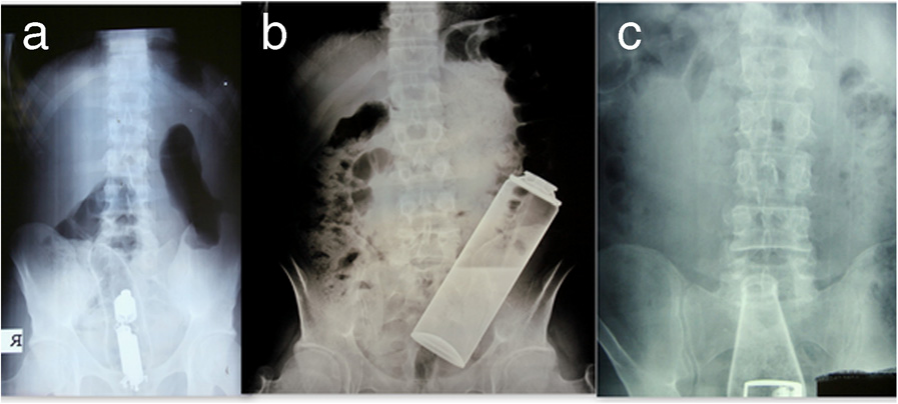
**Abdominal X**-**rays of patients with rectal FB. (a)** Vibrator, **(b)** shaving foam bottle, **(c)** bottle.

**Figure 2:**
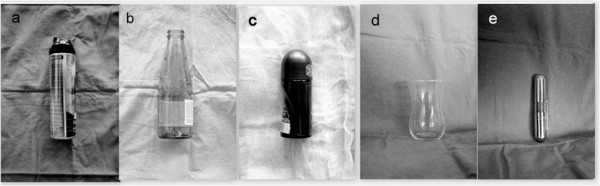
**Photographs of extracted foreign bodies. (a)** shaving foam bottle, **(b)** bottle, **(c)** deodorant, **(d)** glass, **(e)** metal object.

A total of 30 patients, 26 men and 4 women, were admitted with retained rectal foreign body or associated complications. The mean age of the patients were 43 (range, 20–63) years. As for the reason of insertion, 12 patients reported sexual activity, 2 reported an accident in the house and 5 reported that the objects were forcefully introduced into the anus. 11 patients had been unable to state description. Fifteen patients (50%) had high alcohol intake and 3 patients (10%) had psychiatric disease at admission. The most common complaint among the patients was perianal (90%) and abdominal pain (70%). Abdominal X-rays were helpful diagnosis and localization of FB (Figure 1). After the first evaluation in the emergency service, all the patients were hospitalized and evaluation for extraction was carried out in the operating room. Characteristics, localization, type of extraction of foreign bodies were detailed in Table 1. Most of the foreign bodies (23 of 25) were located in the 2/3 distal rectum; remaining 2 FB were located in rectosigmoid junction. Transanal route was the first choice for extraction and it was performed in 23 patients (92%) succesfully. Various surgical techniques such as anal dilatation and digital extraction in 8 (40%) patients, surgical forceps and foley catheters in 10 (50%) patients, and in 2 (10%) patients by means of rectosigmoidoscopy for extraction of rectal FB, have been applied. Figure 2 shows various extracted bodies. Regional anaesthesia was the most common technique for muscle relaxation and it was preferred in 12 (40%) patients. Anal block and intravenous sedation was undertaken in the first 8 (26.6%) and in the remaining 10 (33.4%) patients general anaesthesia was carried out. Seven patients needed emergent laparatomy. Fife of these patients with perforation or severe rectal injury and the remaining 2 patients with failure of transanal extraction. On laparatomy, colotomy, loop colostomy, Hartmann’s procedure and rectal suturation were applied in different patients.

**Table 1:** **Characteristics**, **localization**, **type of extraction of rectal foreign bodies**

	**Patient**	**Transanal extraction**	**Laparatomy**
	**(n=30)**	**(n = 23)**	**(n = 7)**
**Type of foreign body**			
Glass	8	8	1
Bottle	6	5	1
Metal object	5	5	1
Vibrator	2	2	
Toilet Bush	1		1
**Localisation in rectum**			
Proximal (%)	2 (8)	-	2
Distal (%)	23 (92)	23	3
Other*	5		3

*: Patients are free of FB but existence of colorectal injury and history of FB access.

On evaluation with rectal examination and rectosigmoidoscopy, most of rectal injuries (10 patients,%33) are classified as grade I and II. When local treatment was apllied in grade I and II, diverting colostomy was implemented in 2 patients with Grage III injuries (Table 2).

**Table 2:** Type of rectal injuries, treatment and postoperative complications

	**Treatment**			
	**N**	**%**	**Local**	**Colostomy**
**Colorectal injuries**				
Grade I	6	(20)	6	
Grade II	4	(13.3)	4	
Grade III	4	(13.3)	2	2
Perforation	3	(10)		3
**Complication**				
Wound infection	2			
Perianal infection	1			

The patients were hospitalized for 1 to 7 days (median 4 days) postoperatively. On postoperative period 2 patent with wound infection and 1 patient with mild perianal infection was observed.

## Discussion

Rectal FB are seen less commonly in surgical practice especially in emergency department. This phenomenon is most commonly associated with anal eroticisim. Accidental or iatrogenic events, ingestion of animal bones and foreign bodies, psychiatric diseases and drug trafficking are other reasons [4-6]. Foreign bodies that are retained in rectum have various shapes, numbers, and sizes. Amongst the objects encountered are different types such as bottles, cup, glasses, bananas, carrots, vibrators, metal objects, bulbs, pieces of wood and shaving foam cups, etc. [5-7].

After emergency or hospital admission, patients must be evaluated by surgeons with both a detailed history and physical examination. Digital rectal examination is essential. Patient’s complaints usually vary from obscure anal pain and abdominal discomfort and pain, to constipation and anal hemorrhage. Patients can even present with acute abdomen with peritoneal irritation and pelvic sepsis [2,3,8]. The first complaint of 15% of our patients was retained rectal FB. Abdominal X-rays should be undertaken to identify the location, size and the shape of the subject. Chest X-ray should be undertaken to identify the perforation, as there might be free air under the diaphgram. Before admission many of the patients attempted to extract the FB. Unsuccesful attemps are the main reason of delayed hospital admission and rectal FB related complications such as rectal or colonic perforation, peritonitis, perirectal or perianal sepsis [3,9].

Following the diagnosis and to localize the rectal FB, transanal route is the first choice for extraction especially in low lying objects. Before transanal interventions, acute abdomen due to rectal or colonic perforation should be excluded. In various literature attempts to remove FB in the emergency room or at bedside is initially preferred [10,11]. The succes rate of bedside or emergency room attempts are about 16 to 75% in some literatures [12]. Repeated and vigorous efforts to remove rectal FB cause distress, pain and profound involuntary anorectal spazm; it is the main source of this reduced succes rate. In this study all the efforts to extract the rectal FB was carried out in the operating room. Patient personal privacy, Turkish sociocultural assets, and technical and medical requirements cause surgeons to choose this method. In the operating room adequate anesthesia is applied and various instruments are used depending on the foreign bodies characteristics and this improves the nonoperative success rate [12-15]. Adequate anal dilatation by way of caudal or anal block and intravenous sedation is essential for succesful transanal extraction. Sphincter function, tone and contractilitiy and continence should be evaluated. Bimanual pressure on anterior abdominal wall, grasping with forceps, manuplation with foley catheter,magnets for metal objects and rectosigmoidoscopy is complementary techniques for transanal removal of the FB [16]. These methods can be more effectively used only in the operating room.

Prolonged retain period and unsuccessful attempts to remove rectal foreign body by the patient are two important factors that reduce transanal achievement. In our series the success rate of transanal extraction is up to 90 percent. It is related to advantages of operating room and short admission time of our patients. Objects larger than 10cm and those located in the proximal rectum are most likely to require surgical intervention in literature [10]. In our study proximal rectal localization of foreign bodies were more affected laparatomy requirement.

When endoscopic or manual transanal extraction fails or complications are present, laparatomy is necessary [17-19]. Different operative techniques can also be used for the removal of the foreign body and treatment of the complications. The decision to perform colostomy to primary rectal suturing only depends on various factors such as intraabdominal contamination, grade of rectal injury, extend of perianal trauma and chronicity of the case. On laparatomy milking the objects towards the rectum or anus enables the surgeon to extract FB without colotomy. Laparascopic asistance can be used in transanal extraction of proximally migrated FB. It allows for easy removal and direct visualization of the rectum to evaluate for injury. Laparascopic primary suturing, resection and diverting colostomy could be realised [20].

After difficult extraction procedure rectal and distal colonic mucosa is have to evaluate with rectosigmoidoscopy that determine extend of injury and exclude possible perforation. In postextraction rectosigmoidoscopy most of the rectal injuries are in grade I and II as in our series [11].

Surgeons must be aware, in patients with chronicity, of serious anorectal injuries, possibility of perirectal sepcis, and important sequelae such as anal incontinence, fistulas and stenosis in the follow-up period [21].

**Figure 3:**
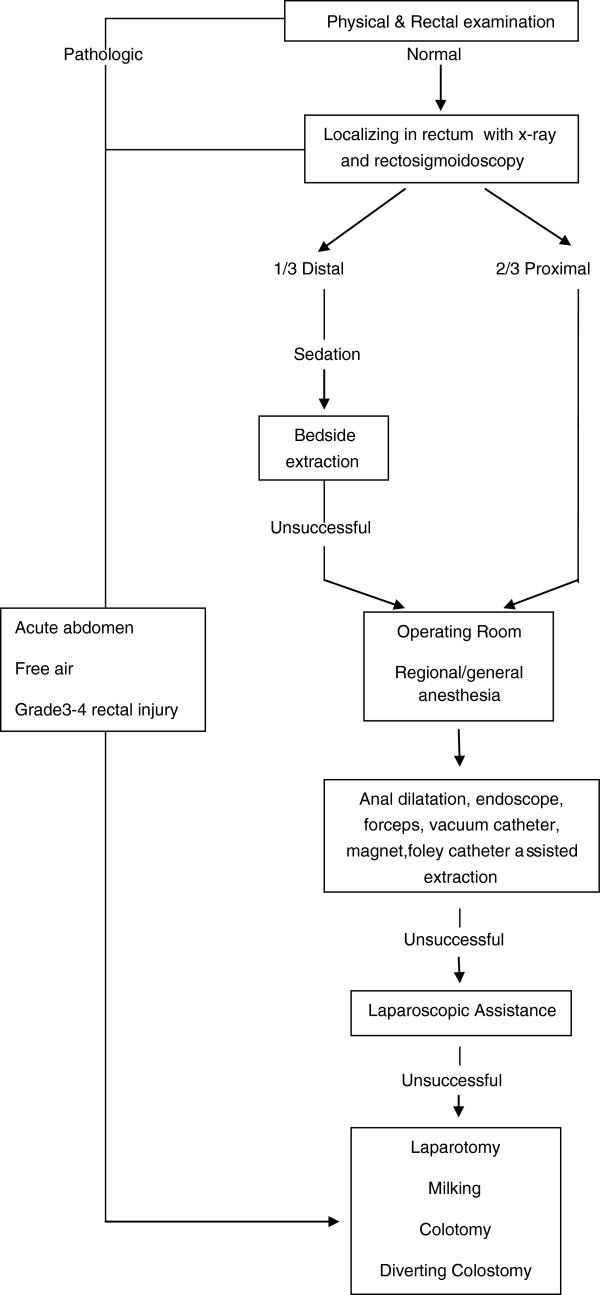
Management algorithm of colorectal foreign body.

Our clinical algorithm was showed in Figure 3. This treatment guide was developed in the light of our clinical experiences. This sequential management system which we use in our clinical practice of colorectal FB, have helped transanal extraction rate to reach over 90%.

All the patients should be evaluated psychologically. Patients presented with foreign bodies in the rectum should be asked for different sexual behaviours such as homosexuality. Most of the patients reject the abnormal sexual activities. Additionally, the patients should be examined for the use of alcohol and narcotic drugs. 50% of our cases reported high level intake of alcoholic beverages before rectal FB introduction.

## Conclusions

Retained rectal foreign bodies are usually related to improper anal sexual behaviour. Patients should be evaluated with a careful physical and rectal examination and plain radiograms for correct diagnosis and localization. Small, low lying rectal foreign bodies may be extracted at bedside and in the emergency room. Forceful and repeated efforts without sphincter relaxation gives rise to proximal migration of objects and unwanted complications such as rectal perforation. The operating room serves appropiate anaesthesia for muscle relaxation and tecnical advantages especially in transanal extraction. If the objects are large and proximally migrated and if the patients suffer from peritonitis due to rectal or colon perforation or pelvic sepsis, laparatomy is performed witout much delay.

## Competing interests

The authors declare that they have no competing interests.

## Authors’ contributions

SYY: conception and design, or acquisition of data, or analysis and interpretation of data, have given final approval of the version to be published. MK: conception and design, or acquisition of data, or analysis and interpretation of data. SA: revising it critically for important intellectual content; AC: revising it critically for important intellectual content; HTT: have made substantial contributions to conception and design. SH: have made substantial contributions to conception and design. All authors read and approved the final manuscript.
